# Sequential Acinetobacter lwoffii Bacteremia and Early Carbapenem-Resistant Acinetobacter baumannii Pneumonia in Diabetic Ketoacidosis: A Case Report

**DOI:** 10.7759/cureus.107628

**Published:** 2026-04-24

**Authors:** Anu Prasad

**Affiliations:** 1 Critical Care Medicine, Tata Main Hospital, Jamshedpur, IND

**Keywords:** acinetobacter lwoffii, antimicrobial resistance, bacteremia, carbapenem‑resistant acinetobacter baumannii (crab), chronic alcohol abuse, diabetic ketoacidosis, mixed acinetobacter infection, opportunistic infection, ventilator‑associated pneumonia

## Abstract

*Acinetobacter lwoffii (A. lwoffii)* is traditionally considered a low-virulence commensal organism, yet it can cause invasive infections in immunocompromised hosts. The sequential appearance of *A. lwoffii *bacteremia with carbapenem-resistant *Acinetobacter baumannii (A. baumannii)* (CRAB) infection in the same patient is rarely reported and presents unique diagnostic and therapeutic challenges. We describe a case of a 51-year-old male with underlying diabetes mellitus and a background of chronic alcohol use who presented with septic shock secondary to bilateral pneumonia, complicated by underlying diabetic ketoacidosis (DKA). Admission blood cultures grew *A. lwoffii* bacteremia with pan-susceptibility except for colistin resistance. Deep endotracheal aspirate cultures later grew extensively drug‑resistant *A. baumannii*. The patient received sequential targeted antimicrobial therapy comprising meropenem for *A. lwoffii* bacteremia with the addition of colistin for CRAB pneumonia, alongside aggressive supportive care, resulting in complete recovery and discharge. This report highlights the pathogenic potential of *A. lwoffii* in diabetic patients with metabolic decompensation and emphasizes the importance of recognizing mixed *Acinetobacter* infections in critically ill patients. Timely microbiological diagnosis and appropriate antimicrobial therapy are essential for favorable outcomes.

## Introduction

*Acinetobacter* species constitute a clinically significant group of pathogens, particularly in ICU settings. These aerobic, non-fermenting, Gram-negative coccobacilli are ubiquitous in the environment and can survive prolonged desiccation, adhere to inanimate surfaces, and contaminate medical equipment, thereby facilitating healthcare-associated transmission [1,2]. Among this genus, *Acinetobacter baumannii* *(A. baumannii)* is well established as a virulent, multidrug-resistant organism responsible for severe nosocomial infections, including ventilator-associated pneumonia (VAP), bloodstream infections, and septic shock [3]. Its pathogenicity is mediated by multiple mechanisms, such as outer membrane protein A (OmpA)-induced apoptosis, biofilm formation on medical devices, iron acquisition systems, and rapid horizontal transfer of resistance determinants [4]. Carbapenem-resistant *A. baumannii* (CRAB) is designated a World Health Organization (WHO) critical priority pathogen, with reported mortality rates ranging from 20% to 60%, with rates particularly high in septic shock or secondary bacteremia [5].

In contrast, *Acinetobacter lwoffii (A. lwoffii)*, traditionally regarded as a commensal colonizing the skin and oropharynx, has increasingly been recognized as an opportunistic pathogen in immunocompromised hosts. In the setting of metabolic stress and invasive illness, *A. lwoffii* has been associated with invasive infections, including bloodstream infections, pneumonia, soft-tissue infections, visceral abscesses, neonatal sepsis, and central nervous system infections following environmental exposure or early healthcare contact [1,6-8]. Notably, *A. lwoffii* is not considered a frequent blood culture contaminant, and its recovery from aerobic blood cultures obtained from separate venipuncture sites, when correlated with compatible clinical findings, supports a diagnosis of true bacteremia [1,6].

Diabetes mellitus, particularly when complicated by diabetic ketoacidosis (DKA), impairs host immune defenses through reduced neutrophil chemotaxis, phagocytosis, and oxidative burst activity, thereby weakening early host defenses and predisposing patients to opportunistic infections [9,10]. Chronic alcohol abuse independently contributes to immune dysregulation and further increases susceptibility to invasive infections caused by organisms of relatively low intrinsic virulence, such as *A. lwoffii* [1,11]. The close temporal occurrence of *A. lwoffii* bacteremia and CRAB pneumonia is uncommon and poses therapeutic challenges due to distinct susceptibility profiles, underscoring the importance of species‑level identification and individualized antimicrobial therapy [1,12,13]. We present this case to highlight these challenges and review current management considerations.

## Case presentation

Patient information and clinical history

A 51‑year‑old male presented to the emergency department with a two-to-three‑day history of low‑grade fever and dry cough, accompanied by progressive dyspnoea and generalized weakness since the morning of presentation. He had received treatment at another healthcare facility before referral; however, details regarding diagnostic evaluations or antimicrobial therapy during that period were unavailable. His medical history was significant for poorly controlled type 2 diabetes mellitus and hypertension. He resided in an urban, non‑crowded household and denied recent travel, sick contacts, or occupational exposure to dust, chemicals, agricultural environments, or healthcare settings. He reported a history of chronic alcohol consumption, currently reduced to occasional intake, and denied tobacco use or illicit drug abuse. There was no history of corticosteroid use, malignancy, organ transplantation, or known HIV infection, and no other recognized immunosuppressive condition was identified.

Initial assessment

On arrival (Day one), the patient was conscious, oriented, and in mild respiratory distress. He had sinus tachycardia (heart rate 120/min), blood pressure 137/80 mmHg, respiratory rate 26/min, and oxygen saturation of 98% on room air. Capillary blood glucose was 348 mg/dL. Arterial blood gas (ABG) analysis demonstrated severe metabolic acidosis with appropriate respiratory compensation (pH 7.10, PaCO₂ 8 mmHg, bicarbonate 6.7 mmol/L, base excess −26.4 mmol/L). Serum lactate levels were within the normal range, excluding lactic acidosis as a contributing factor. Urinalysis revealed strongly positive ketonuria (4+), confirming the diagnosis of DKA.

Initial management

**Figure 1:**
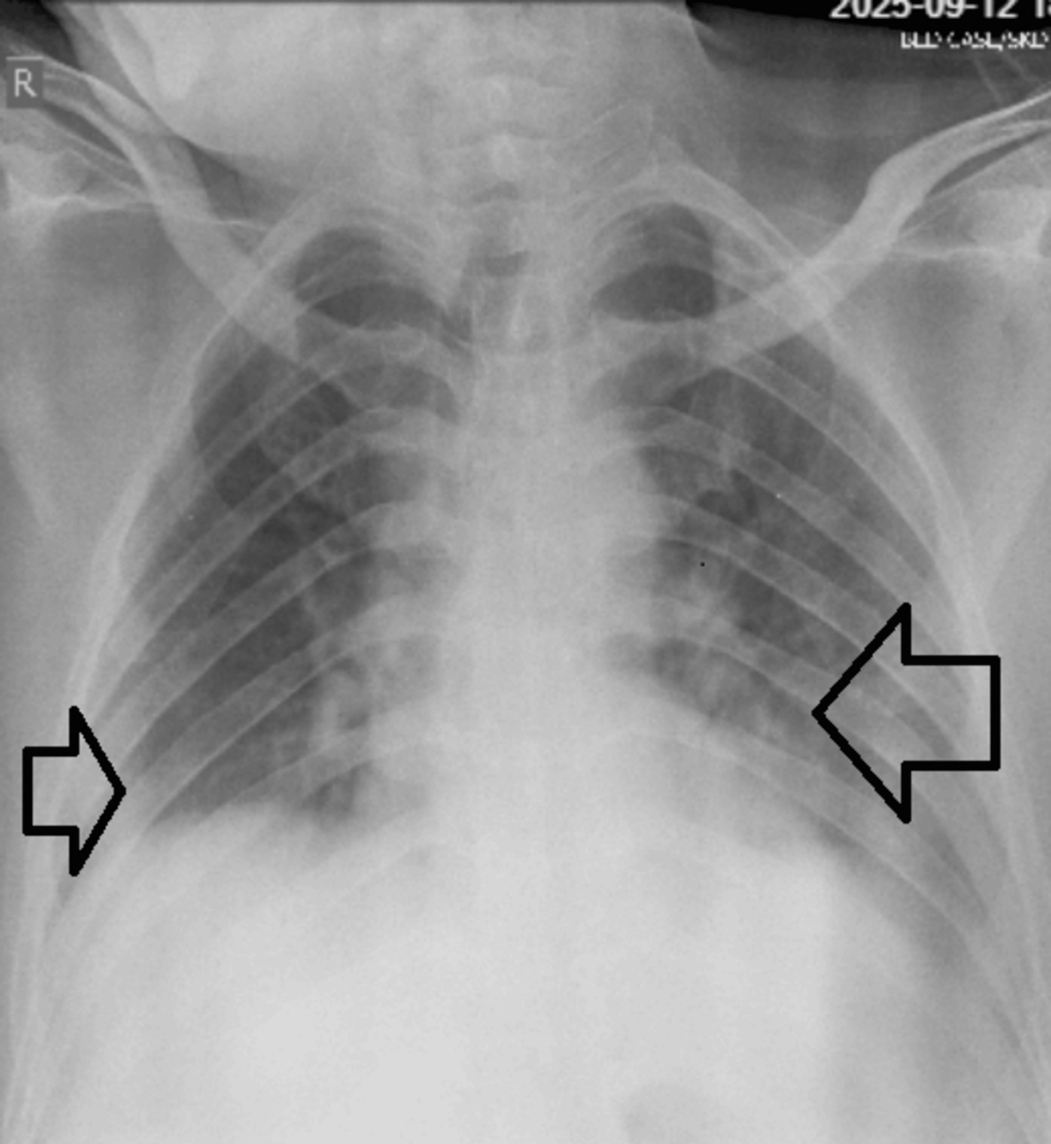
Bedside chest radiograph on Day 1 The image shows patchy, ill-defined opacity in the right lower zone adjacent to the diaphragm and another ill-defined left perihilar haziness spreading to the mid-lower zone, showing bilateral pneumonia (arrows)

Initial management in the emergency department included intravenous fluid resuscitation, initiation of a continuous insulin infusion per institutional protocol, and administration of intravenous sodium bicarbonate (100 mEq) due to severe acidemia. A chest radiograph was obtained in view of respiratory distress, which revealed patchy, ill‑defined opacities in both lungs, consistent with bilateral pneumonia (Figure 1). In view of the observed pulmonary involvement and the potential for respiratory compromise in the context of severe metabolic derangement, supplemental oxygen was initiated. The patient was subsequently admitted to the ICU for close monitoring and further management.

Differential diagnoses at presentation included DKA with sepsis‑related metabolic acidosis, infection‑related respiratory failure (community‑acquired or aspiration pneumonia), alcoholic ketoacidosis, and septic shock. The constellation of profound hyperglycemia, severe metabolic acidosis, and marked ketonuria supported DKA as the primary diagnosis.

Within hours of ICU admission (Day one), the patient developed worsening respiratory distress requiring emergent endotracheal intubation and mechanical ventilation. Hemodynamic instability necessitated vasopressor support with norepinephrine (0.1 μg/kg/min), along with continued fluid resuscitation. As part of the sepsis evaluation, blood, deep endotracheal aspirate, and urine samples were obtained for microbiological culture, in addition to routine laboratory investigations. Empirical antimicrobial therapy with intravenous piperacillin-tazobactam (4.5 g every eight hours) and moxifloxacin (400 mg once daily) was initiated from Day one for severe pneumonia in the context of DKA, in accordance with institutional guidelines.

Persistent severe acidaemia on repeat ABG analysis prompted the initiation of a continuous sodium bicarbonate infusion. Serial ABG monitoring demonstrated significant improvement in acid-base parameters, following which bicarbonate therapy was discontinued. The serial ABG reports are summarized in Table 1.

**Table 1:** Summary of arterial blood gas analysis during initial resuscitation in the ICU ICU: intensive care unit; pH: potential of hydrogen; PaCO₂: partial pressure of arterial carbon dioxide; PaO₂: partial pressure of arterial oxygen; Hct: hematocrit; SaO₂: arterial oxygen saturation; HCO₃⁻: bicarbonate; BE: base excess; FiO₂: fraction of inspired oxygen; PEEP: positive end‑expiratory pressure

Variables	On admission	After 2 hours	After 6 hours	After 12 hours	After 18 hours	Reference values
pH	7.1	7.15	7.18	7.42	7.43	7.35-7.45
PaCO_2_ (mmHg)	8	11.6	27.1	22.1	23.7	35.0-45.0
PaO_2_ (mmHg)	129	107	89	245	139	80-100
Hct (%)	45.9	43.7	40.4	38.4	36.2	--
SaO_2_ (%)	96.1	95.6	92.4	98	97.2	95-100
HCO₃⁻ (mmol/L)	6.7	8.1	11.2	17.9	18.7	--
BE (mmo/L)	-26.4	-23.8	-17.1	-9.4	-8.1	--
Lactate (mmol/L)	0.7	0.9	0.9	1	0.9	0.5-1.5
Anion gap (mmol/L)	9.4	8.4	3.1	-2.3	0.8	--
Sodium (mmol/L) [corrected]	126	132	138	132	138	135-145
Potassium (mmol/L)	5.3	5	4.4	4.2	3.4	3.5-5.5
Blood glucose (mg/dL)	348	294	145	183	132	65-95
FiO_2_ (%)	On ambient air	60	60	35	40	--
PEEP (mmHg)	NA	8	8	8	8	--

Laboratory investigations

Laboratory evaluation revealed moderate leukocytosis with neutrophilia, preserved hemoglobin and platelet counts, mildly elevated urea, and liver enzymes within normal limits except for a mild elevation in alkaline phosphatase. Inflammatory markers, including C‑reactive protein and procalcitonin, were markedly elevated. Chronic glycemic control was poor (HbA1c 9.7%), and coagulation studies showed mildly prolonged prothrombin time. The serial laboratory findings are summarized in Table 2.

**Table 2:** Serial laboratory findings TLC: total leukocyte count; ALT: alanine aminotransferase; AST: aspartate aminotransferase; ALP: alkaline phosphatase; CRP: C‑reactive protein; PT: prothrombin time; INR: international normalized ratio; aPTT: activated partial thromboplastin time; HbA1c: glycated hemoglobin

Laboratory parameters	Values (Day 1)	Values (Day 5)	Unit of measurement	Reference range
Hemoglobin	14.1	10.3	g/dL	11.5-16.5
TLC	17300	9420	Per cumm	4000-11000
Differential count (neutrophil %)	81	74	%	60-70
Platelets	275000	164000	Per cumm	150000-450000
Urea	76.7	26.2	mg/dL	15-40
Creatinine	1.33	0.91	mg/dL	0.5-1.5
Sodium	134	144	mEq/L	136-146
Potassium	5.3	4.1	mEq/L	3.5-5.5
Chloride	106	112	mEq/L	95-108
Total bilirubin	0.53	0.83	mg/dL	0.2-1.0
ALT	24.4	38.4	U/L	0-45
AST	41.3	88.5	U/L	0-35
ALP	206.8	120.3	U/L	53-141
Albumin	3.8	2.76	gm/dl	3.5-5.2
CRP	6.65	6.76	mg/dL	0.08-0.79
Procalcitonin	15.61	3.21	ng/mL	0.02-0.3
PT	17.9	16.8	Seconds	12.0-14.9
INR	1.35	1.26	--	0.8-1.2
aPTT	28.1	28.5	Seconds	28.1-31.7
Fibrinogen	530	457	mg/dL	200-400
HbA1C	9.7	NA	%	04-Jun
Urine ketones	2+	Absent	--	Negative-trace

Clinical course

The patient remained mechanically ventilated (FiO₂ 0.4, PEEP 8 cm H₂O) and required norepinephrine titrated between 0.1 and 0.4 μg/kg/min to maintain a mean arterial pressure >65 mmHg. Sedation was maintained at a Richmond Agitation-Sedation Scale score of 0 to −2. Strict intake-output monitoring was ensured, with adequate urine output maintained and an overall neutral fluid balance achieved. Urine ketones remained positive for three days before resolving. Supportive care included continuous insulin infusion, thiamine supplementation, early enteral nutrition, and lung‑protective ventilation strategies.

Microbiological findings

Microbiological evaluation revealed no growth from the initial endotracheal aspirate. Two aerobic blood culture bottles collected from separate venipuncture sites on Day one turned positive at 48 hours, yielding *A. lwoffii.* The isolate from both bottles was susceptible to carbapenems, fluoroquinolones, third‑generation cephalosporins, aminoglycosides, and cefoperazone-sulbactam, but resistant to piperacillin-tazobactam and colistin. In light of confirmed bacteremia and critical illness, definitive therapy with intravenous meropenem (loading dose 2 g, followed by 1 g every eight hours) was initiated.

A repeat endotracheal aspirate obtained on Day three grew carbapenem‑resistant *A. baumannii*, susceptible only to colistin. Intravenous colistin was therefore added (loading dose 9 million IU, followed by 4.5 million IU every 12 hours). The antibiotic susceptibility profile is summarized in Table 3.

**Table 3:** Antimicrobial susceptibility profiles of Acinetobacter lwoffii (blood culture on Day 1) and Acinetobacter baumannii (deep tracheal aspirate culture on Day 3) MIC breakpoints interpreted according to CLSI guidelines. Colistin interpreted as per EUCAST recommendations. TMP-SMX values are expressed as trimethoprim/sulfamethoxazole; laboratory-reported value (≤ 20 mcg/mL) corresponds to susceptible range MIC: minimum inhibitory concentration; TMP/SMX: trimethoprim/sulfamethoxazole; S: sensitive; I: intermediate; R: resistant; CLSI: Clinical and Laboratory Standards Institute; EUCAST: European Committee on Antimicrobial Susceptibility Testing

Antibiotic	MIC value (mcg/mL)	Reference MIC (CLSI/EUCAST)	Interpretation
*Acinetobacter lwoffii* (blood culture): colony counts were not reported			
Amikacin	≤ 2 mcg/mL	S ≤ 16, I = 32, R ≥ 64	S
Cefepime	0.25 mcg/mL	S ≤ 8, I = 16, R ≥ 32	S
Cefoperazone/sulbactam	≤ 8 mcg/mL	S ≤ 16, I = 32, R ≥ 64	S
Ceftriaxone	2 mcg/mL	S ≤ 8, I = 16, R ≥ 32	S
Ciprofloxacin	≤ 0.06 mcg/mL	S ≤ 1, I = 2, R ≥ 4	S
Colistin (EUCAST)	≥ 16 mcg/mL	S ≤ 2, R > 2 (EUCAST)	R
Gentamicin	≤ 1 mcg/mL	S ≤ 4, I = 8, R ≥ 16	S
Imipenem	≤ 0.5 mcg/mL	S ≤ 2, I = 4, R ≥ 8	S
Meropenem	≤ 0.25 mcg/mL	S ≤ 2, I = 4, R ≥ 8	S
Piperacillin/tazobactam	≥ 128 mcg/mL	S ≤ 16, I = 32-64, R ≥ 128	R
TMP/SMX	≤ 20 mcg/mL	S ≤ 2/38, R ≥ 4/76	S
*Acinetobacter baumannii* (deep tracheal aspirate culture) colony counts: ≥10⁵ CFU/mL			
Amikacin	≥ 64 mcg/mL	S ≤ 16, I = 32, R ≥ 64	R
Cefepime	≥ 32 mcg/mL	S ≤ 8, I = 16, R ≥ 32	R
Cefoperazone/sulbactam	≥ 64 mcg/mL	S ≤ 16, I = 32, R ≥ 64	R
Ceftazidime	≥ 64 mcg/mL	S ≤ 8, I = 16, R ≥ 32	R
Ciprofloxacin	≥ 4 mcg/mL	S ≤ 1, I = 2, R ≥ 4	R
Colistin	2 mcg/mL	S ≤ 2, R > 2 (EUCAST)	S
Gentamicin	≥ 16 mcg/mL	S ≤ 4, I = 8, R ≥ 16	R
Imipenem	≥ 16 mcg/mL	S ≤ 2, I = 4, R ≥ 8	R
Levofloxacin	≥ 8 mcg/mL	S ≤ 2, I = 4, R ≥ 8	R
Meropenem	≥ 16 mcg/mL	S ≤ 2, I = 4, R ≥ 8	R
Minocycline	16 mcg/mL	S ≤ 4, I = 8, R ≥ 16	R
Piperacillin/tazobactam	≥ 128 mcg/mL	S ≤ 16, I = 32-64, R ≥ 128	R
TMP/SMX	≥ 320 mcg/mL	S ≤ 2/38, R ≥ 4/76	R

Outcome and follow-up

**Figure 2:**
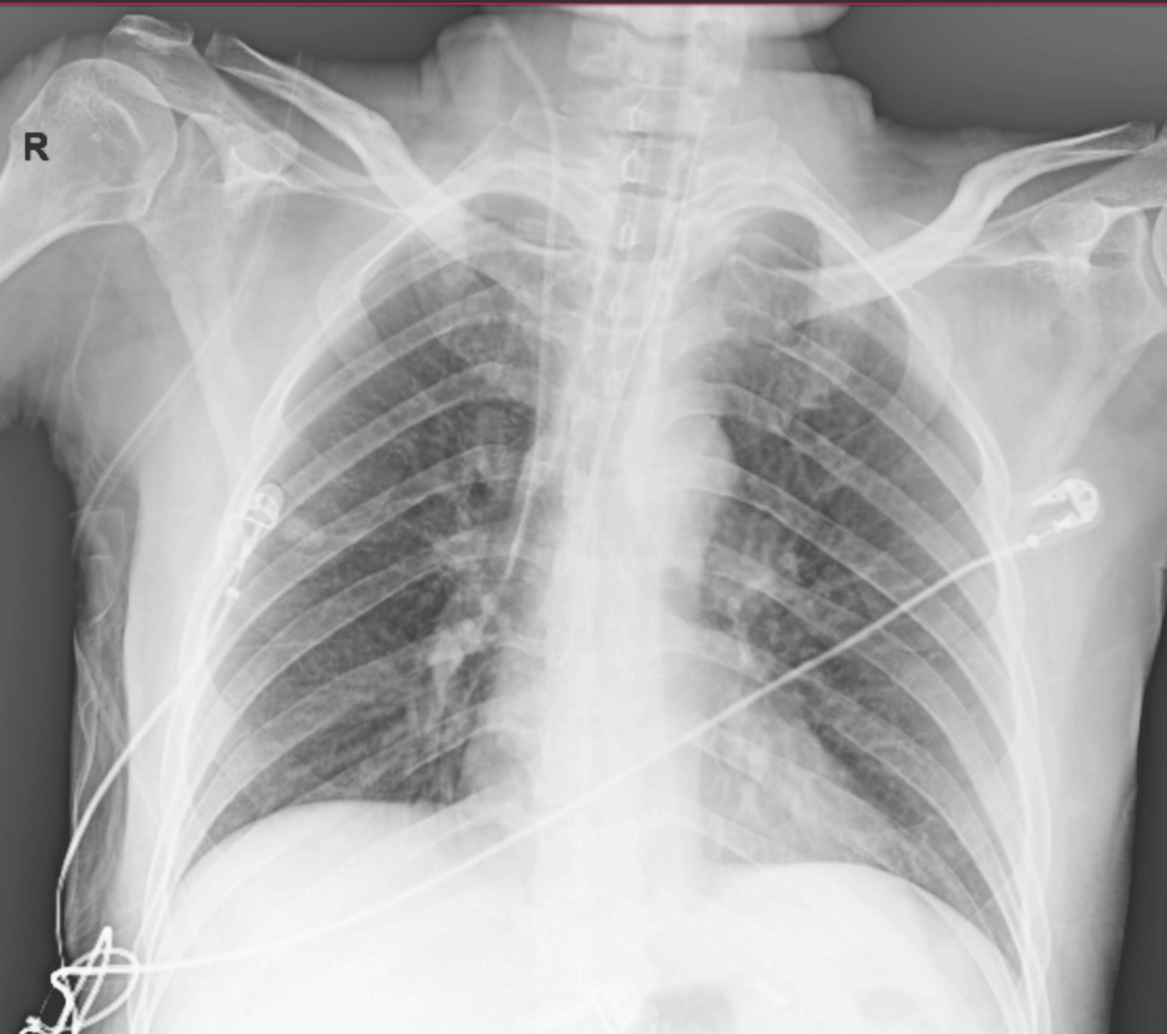
Bedside chest radiograph on Day 5 The image shows reduction in bilateral air-space opacities with improved lung aeration, suggestive of resolving pneumonia

Repeat blood cultures obtained on Day five were sterile, confirming the clearance of bacteremia, and procalcitonin levels decreased by approximately 80%. Chest imaging on Day five demonstrated radiographic improvement (Figure 2). Daily spontaneous breathing trials were initiated, leading to successful extubation on Day seven. Vasopressors were discontinued on Day eight, and the patient was transferred to the general medical ward.

With continued clinical improvement and microbiological clearance, antimicrobial therapy was de‑escalated before discharge in accordance with culture sensitivities and antimicrobial stewardship principles. The patient was discharged home in stable condition on Day 11 with appropriate follow‑up.

## Discussion

This report highlights key clinical principles related to *Acinetobacter* infections in immunocompromised hosts. The close temporal occurrence of *A. lwoffii* bacteremia followed by CRAB respiratory infection reflects a complex interplay between host immune dysfunction and microbial pathogenicity in the setting of metabolic stress.

*Acinetobacter lwoffii* as an emerging pathogen

Although *A. lwoffii* is traditionally regarded as a commensal colonizing the skin and oropharynx, it has increasingly been recognized as a cause of invasive disease in vulnerable populations. Reported manifestations include pyogenic liver abscesses in patients with diabetes, cellulitis in those with combined immunosuppression, and catheter‑related bloodstream infections [7,8]. The pathogenic potential of *A. lwoffii* appears closely linked to host immune status. In this patient, poorly controlled diabetes (HbA1c 9.7%) complicated by DKA likely resulted in impaired innate immune defenses secondary to metabolic derangements [14], potentially facilitating progression from colonization to invasive bloodstream infection.

Pediatric series further suggest that *A. lwoffii* bacteremia generally demonstrates favorable antimicrobial susceptibility patterns and good outcomes when appropriately treated [15]. Direct evidence of prior colonization was unavailable, as no antecedent surveillance or non‑sterile site cultures had been obtained. Acquisition may have occurred through transient endogenous carriage, environmental exposure, or early healthcare contact, particularly in the presence of metabolic stress and invasive illness [1]. The absence of additional immunosuppressive conditions - such as corticosteroid use, malignancy, transplantation, or HIV infection - supports the central role of metabolic decompensation.

Diabetic ketoacidosis and bacterial infections

Diabetes mellitus, particularly when complicated by DKA, is a recognized risk factor for severe bacterial infections. Infection precipitates approximately one‑third to one‑half of DKA episodes, most commonly pneumonia and urinary tract infections [16,17]. Conversely, DKA can worsen infections through dehydration, acidosis, and impaired leukocyte function, creating a reinforcing cycle of infection and metabolic instability [14,17]. Procalcitonin has utility as an adjunctive marker of bacterial infection in DKA when interpreted alongside clinical and microbiological data [16,17]. In this case, markedly elevated procalcitonin levels with an approximately 80% decline after antimicrobial initiation supported a significant bacterial contribution and correlated with clinical improvement. While diabetes and DKA are associated with increased susceptibility to infection due to transient immune dysfunction, no specific epidemiologic association between diabetes and *Acinetobacter* infections has been established; the organisms noted in this case are best interpreted as opportunistic pathogens in a critically ill host.

Impact of chronic alcohol use

Chronic alcohol abuse likely compounded susceptibility and disease severity in this patient. Alcohol‑related immune dysfunction, including impaired neutrophil and macrophage function and suppression of adaptive immunity, predisposes to severe infections. In conjunction with the metabolic and inflammatory stress of DKA, this impairment may have facilitated both *A. lwoffii *bacteremia and CRAB pneumonia [11].

Carbapenem‑resistant *Acinetobacter baumannii*

CRAB represents a major therapeutic challenge in modern clinical practice, particularly in critically ill patients. The organism’s clinical success is driven by its ability to persist in hospital environments and its capacity to evade host defenses through a combination of surface adherence, biofilm development, membrane‑associated injury mechanisms, and efficient resistance determinants, including efflux pumps and β‑lactamase production [4]. These characteristics have contributed to its categorization as a World Health Organization critical‑priority pathogen, with infections associated with substantial morbidity, prolonged hospitalization, and high mortality rates [5].

Given the limited efficacy of monotherapy, management of severe CRAB infections increasingly relies on combination antimicrobial strategies. Contemporary guidelines advocate individualized regimens incorporating polymyxins, tigecycline, high‑dose sulbactam‑containing agents, or cefiderocol when available, with selection guided by infection severity, site, and susceptibility patterns [18]. However, optimal therapy remains an area of ongoing investigation.

Unlike many β‑lactamase inhibitors, sulbactam has direct antimicrobial activity against *A. baumannii*, mediated through high‑affinity binding to penicillin‑binding proteins, particularly PBP2 [13,18]. High‑dose ampicillin-sulbactam has therefore emerged as a cornerstone agent in the treatment of CRAB, either alone when susceptible or more commonly in combination regimens. Clinical studies and observational data support the use of ampicillin-sulbactam combined with meropenem and/or polymyxins in severe infections, with these regimens demonstrating improved microbiological clearance and, in some settings, better clinical outcomes compared with polymyxin monotherapy [13,18].

The development of sulbactam-durlobactam represents a significant advance in this therapeutic landscape. Durlobactam is a novel β‑lactamase inhibitor targeting class A, C, and D enzymes, thereby protecting sulbactam from hydrolysis and restoring activity against resistant *A. baumannii* strains. In the ATTACK phase 3 trial, sulbactam-durlobactam demonstrated non‑inferiority to colistin for serious *A. baumannii* infections, with significantly reduced nephrotoxicity, positioning it as a preferred option where accessible [19]. Despite its promise, real‑world availability remains limited in many regions.

In the present case, the CRAB isolate exhibited susceptibility only to colistin (MIC 2 µg/mL), necessitating the use of a polymyxin‑based regimen. Although polymyxin B may offer pharmacokinetic advantages over colistimethate sodium, treatment choice reflected institutional laboratory practices, formulary availability, and susceptibility reporting [20]. The favorable clinical response observed underscores the importance of early pathogen identification, resistance profiling, and timely initiation of directed therapy when managing CRAB infections.

Mixed *Acinetobacter* infections and therapeutic implications

Infection involving more than one *Acinetobacter* species is infrequently reported but remains biologically plausible given shared environmental niches and hospital reservoirs [1,12]. *A. lwoffii* is often dismissed as a contaminant owing to its commensal nature and lower virulence profile. Here, however, its isolation from two aerobic blood cultures obtained at admission supported true Gram‑negative bacteremia. The subsequent recovery of CRAB from a deep endotracheal aspirate on Day three was consistent with a temporally distinct nosocomial respiratory infection.

The differing resistance profiles of the two organisms presented therapeutic challenges, with meropenem providing effective coverage for *A. lwoffii* bacteremia and colistin required for CRAB pneumonia. Early recognition of both infections, timely culture‑directed therapy, and ongoing reassessment contributed to the favorable clinical outcome.

Prognostic factors and clinical outcomes

Several factors contributed to the favorable outcome observed in this patient, including early recognition and aggressive management of DKA, prompt initiation of broad‑spectrum antimicrobials with subsequent culture‑guided adjustment, adequate supportive ICU care, and close microbiological monitoring. Clearance of bacteremia was confirmed by sterile repeat blood cultures, and declining procalcitonin levels supported therapeutic response. Together, these measures highlight the value of timely diagnosis, antimicrobial stewardship, and individualized management in critically ill patients with mixed *Acinetobacter* infections. The novelty of this case lies in the sequential occurrence of two clinically distinct *Acinetobacter* infections with divergent antimicrobial susceptibility patterns in a patient with DKA.

## Conclusions

The sequential occurrence of *A. lwoffii *bacteremia followed by CRAB respiratory infection in a patient with DKA highlights the complex interplay between diabetes‑related immune dysfunction and invasive *Acinetobacter* disease. The additional history of chronic alcohol abuse likely compounded immune impairment and metabolic stress, further predisposing the patient to opportunistic infections. This report underscores the pathogenic potential of typically commensal *Acinetobacter* species in metabolically decompensated hosts and illustrates the therapeutic challenges posed by mixed infections involving organisms with markedly divergent antimicrobial resistance profiles. Clinicians should maintain a high index of suspicion for both common and uncommon *Acinetobacter* species in critically ill patients with diabetes presenting with sepsis. Prompt microbiological identification, comprehensive antimicrobial susceptibility testing, and timely initiation of targeted therapy - balanced with antimicrobial stewardship principles - are essential for optimizing clinical outcomes in such complex infections.

## References

[1] Wong D, Nielsen TB, Bonomo RA, Pantapalangkoor P, Luna B, Spellberg B (2017). Clinical and pathophysiological overview of Acinetobacter infections: a century of challenges. Clin Microbiol Rev.

[2] Peleg AY, Seifert H, Paterson DL (2008). Acinetobacter baumannii: emergence of a successful pathogen. Clin Microbiol Rev.

[3] Dijkshoorn L, Nemec A, Seifert H (2007). An increasing threat in hospitals: multidrug-resistant Acinetobacter baumannii. Nat Rev Microbiol.

[4] Morris FC, Dexter C, Kostoulias X, Uddin MI, Peleg AY (2019). The mechanisms of disease caused by Acinetobacter baumannii. Front Microbiol.

[5] Dubey V, Reza N, Hope W (2025). Drug-resistant Acinetobacter baumannii: mortality, emerging treatments, and future pharmacological targets for a WHO priority pathogen. Clin Microbiol Rev.

[6] Ku SC, Hsueh PR, Yang PC, Luh KT (2000). Clinical and microbiological characteristics of bacteremia caused by Acinetobacter lwoffii. Eur J Clin Microbiol Infect Dis.

[7] Singh NP, Sagar T, Nirmal K, Kaur IR (2016). Pyogenic liver abscess caused by Acinetobacter lwoffii: a case report. J Clin Diagn Res.

[8] Sakakiyama M, Hayashi K, Matsuda H, Hayashi M (2025). Acinetobacter lwoffii cellulitis in an immunocompromised patient with decompensated cirrhosis, diabetes mellitus, and psoriasis vulgaris: a case report. Cureus.

[9] Dowey R, Iqbal A, Heller SR, Sabroe I, Prince LR (2021). A bittersweet response to infection in diabetes; targeting neutrophils to modify inflammation and improve host immunity. Front Immunol.

[10] Eledrisi MS, Elzouki AN (2020). Management of diabetic ketoacidosis in adults: a narrative review. Saudi J Med Med Sci.

[11] Sarkar D, Jung MK, Wang HJ (2015). Alcohol and the immune system. Alcohol Res.

[12] Rosa R, Mills J, Munoz Price LS (2015). Clinical and microbiological characteristics of Acinetobacter lwoffii bacteremia compared with Acinetobacter baumannii. Open Forum Infect Dis.

[13] Piperaki ET, Tzouvelekis LS, Miriagou V, Daikos GL (2019). Carbapenem-resistant Acinetobacter baumannii: in pursuit of an effective treatment. Clin Microbiol Infect.

[14] Dhatariya KK, Glaser NS, Codner E, Umpierrez GE (2020). Diabetic ketoacidosis. Nat Rev Dis Primers.

[15] Güneş Ö, Özkaya-Parlakay A, Güney AY (2025). Clinical features, antimicrobial susceptibilities, treatment characteristics and outcomes of paediatric Acinetobacter lwoffii bacteremia: a case series. Eur J Clin Microbiol Infect Dis.

[16] Blanchard F, Charbit J, Van der Meersch G (2020). Early sepsis markers in patients admitted to intensive care unit with moderate-to-severe diabetic ketoacidosis. Ann Intensive Care.

[17] Hao Y, Yang L, Meng X, Tang Y, Wang L (2025). Identification of early predictors and model for bacterial infection in diabetic ketoacidosis patients: a retrospective study. PLoS One.

[18] Tamma PD, Heil EL, Justo JA (2024). Infectious Diseases Society of America 2024 guidance on the treatment of antimicrobial-resistant Gram-negative infections. Clin Infect Dis.

[19] Kaye KS, Shorr AF, Wunderink RG (2023). Efficacy and safety of sulbactam-durlobactam versus colistin for the treatment of patients with serious infections caused by Acinetobacter baumannii-calcoaceticus complex: a multicentre, randomised, active-controlled, phase 3, non-inferiority clinical trial (ATTACK). Lancet Infect Dis.

[20] Zavascki AP, Nation RL (2017). Nephrotoxicity of polymyxins: is there any difference between colistimethate and polymyxin B?. Antimicrob Agents Chemother.

